# Habituation and Dishabituation in Motor Behavior: Experiment and Neural Dynamic Model

**DOI:** 10.3389/fpsyg.2022.717669

**Published:** 2022-04-08

**Authors:** Sophie Aerdker, Jing Feng, Gregor Schöner

**Affiliations:** ^1^Institute for Neural Computation, Ruhr University Bochum, Bochum, Germany; ^2^Motion Analysis Center, Shriners Hospitals for Children, Portland, OR, United States

**Keywords:** habituation, perseveration, neural dynamic model, Dynamic Field Theory, exploration-exploitation

## Abstract

Does motor behavior early in development have the same signatures of habituation, dishabituation, and Spencer-Thompson dishabituation known from infant perception and cognition? And do these signatures explain the choice preferences in A not B motor decision tasks? We provide new empirical evidence that gives an affirmative answer to the first question together with a unified neural dynamic model that gives an affirmative answer to the second question.In the perceptual and cognitive domains, habituation is the weakening of an orientation response to a stimulus over perceptual experience. Switching to a novel stimulus leads to dishabituation, the re-establishment of the orientation response. In Spencer-Thompson dishabituation, the renewed orientation response transfers to the original (familiar) stimulus. The change in orientation responses over perceptual experience explains infants' behavior in preferential looking tasks: Familiarity preference (looking longer at familiar than at novel stimuli) early during exposure and novelty preference (looking longer at novel than at familiar stimuli) late during exposure. In the motor domain, perseveration in the A not B task could be interpreted as a form of familiarity preference. There are hints that this preference reverses after enough experience with the familiar movement. We provide a unified account for habituation and patterns of preferential selection in which neural dynamic fields generate perceptual or motor representations. The build-up of activation in excitatory fields leads to familiarity preference, the build-up of activation in inhibitory fields leads to novelty preference. We show that the model accounts for the new experimental evidence for motor habituation, but is also compatible with earlier accounts for perceptual habituation and motor perseveration. We discuss how excitatory and inhibitory memory traces may regulate exploration and exploitation for both orientation to objects and motor behaviors.

## 1. Introduction

Most behavior is directed at objects in the world that are perceived based on sensory information. Once a particular object has been selected as the target of an action, other objects may effectively become distractors. A selected action must be stabilized against competing actions directed at these other objects. In the development of object-directed action, perseverative reaching may be viewed as a signature of such stabilization (Smith et al., 1999; Thelen et al., 2001). In the classical A not B paradigm (Wellman et al., 1986), infants repeatedly reach for a toy that is hidden at one of two locations, typically two troughs cut out from a box and covered by lids. On each trial, the infant watches as the experimenter hides the toy at the A location and then, after a short delay, pushes the box into the infant's reaching space. After the infant reaches, and typically retrieves the toy, the experimenter gently wrings the toy out of the infant's hand, pulls the box back to its starting position and starts another trial. After six such “A trials,” the toy is next hidden at the B location. Young infants (from around 7 to 10 months of age) then typically perseverate, reaching again for the A location rather than retrieving the toy at the B location. In a sense, they stabilize the reach to A, suppressing the distractor cue to B. Older infants do not make this perseverative error. They are able to follow the cue and switch to the B location.

Habituation is commonly observed in paradigms that probe infant perception and cognition (Colombo and Mitchell, 2009). In a typical visual habituation paradigm a salient visual stimulus is presented to an infant against a nondescript background. Infants' orientation response is measured through “looking time” (total duration of fixation on the stimulus) or by physiological measures such as increased heart rate or sucking frequency. Presentation is repeated, often in a manner that depends on the infants response. A trial starts once the infant looks at the habituation stimulus and may last a fixed maximal duration or may end earlier as soon as the infant looks away from the stimulus. To start a new trial, the renewed presentation of the stimulus is often preceded or accompanied by an attention grabbing stimulus, like a flashing light or a sound effect. Across trials, infants' orientation responses weaken. Habituation trials are repeated during the habituation phase until a criterion is met. Typically, total looking time across three consecutive trials must fall below half the total looking time on the first three habituation trials for the habituation phase to end. In the subsequent test phase new stimuli are presented. Renewed orientation behavior toward such new stimuli is referred to as dishabituation and indicates that habituation is specific to the habituation stimulus. Sometimes, an orientation response continues to be observed when the habituation stimulus is then again presented, a phenomenon referred to as Spencer-Thompson dishabituation (Thompson and Spencer, 1966).

Conceptually, habituation could be viewed as a signature of destabilization where the reduced looking time results from reduced stabilization of visual fixation or, generally, reduced responsiveness (Balkenius, 2000; Sirois and Mareschal, 2002, 2004; Schöner and Thelen, 2006). This is consistent with how habituation manifests itself in preferential looking tasks (Roder et al., 2000) that probe perception in a way that is analogous to how motor decisions are probed in the A not B task. Stimuli at two spatial locations are repeatedly presented to infants. At one location, the stimulus remains the same across repetitions, at the other location it varies and is thus always new to the infant. Orientation is assessed by looking time at either of the two stimuli. Across the first few repetitions, infants tend to look longer at the invariant stimulus, a finding referred to as familiarity preference. After longer exposure, infants tend to look longer at the novel stimulus, a finding referred to as novelty preference.

Familiarity preference may then be viewed as a form of stabilization in which the established spatial orientation resists change to the location of the novel stimulus. Novelty preference would then reflect habituation to the familiar stimulus which destabilizes the orientation response to that stimulus. The pattern of early familiarity and late novelty preference would thus suggest that stabilization predominates early during such repeated stimulation, while destabilization prevails later.

This is how neural dynamic models provide theoretical accounts for both perseverative reaching (Thelen et al., 2001; Dineva and Schöner, 2018) and visual habituation (Schöner and Thelen, 2006; Perone and Spencer, 2013b). Neurons tuned to relevant features are modeled at the population level as neural dynamic fields that span the feature dimensions. Localized activation patterns (or peaks) in these fields represent perceptual or motor states. Activation peaks are induced by external input. Once activation exceeds the threshold of neural transmission, a pattern of recurrent, locally excitatory connectivity within the fields begins to stabilize localized activation peaks. Inhibitory recurrent connectivity, neurophysiologically mediated by a field of inhibitory interneurons, supports selective activation at one field location when multiple locations receive input. Once a peak has been induced, activation in both excitatory and inhibitory populations may be strengthened over time due to a simple learning mechanism, modeled as a memory trace. This accounts for effects across multiple presentations or reaches in these models [and corresponds to the “latent memory trace” in the alternative connectionist model of perseverative reaching (Munakata, 1998)].

In the account for perseverative reaching in the A not B paradigm (Thelen et al., 2001; Dineva and Schöner, 2018), the activation field spans the direction of the infants reaching movements. When a reach to the A location is cued on an A trial, input is provided to the location of the field that corresponds to reaches to that location. Once activation at that field location passes the threshold, a reach to A is predicted. The memory trace of the activation field strengthens activation at that location, making it easier to elicit the same movement again on the next trial. This build-up of a memory trace across trials is responsible for perseveration when the B location is cued on a later B trial. Essentially, the reinforced activation pattern for a reach to the A location competes with activation induced by the cue for a reach to the B location. That induced activation decays over a delay, while the memory trace persists, so that the competition is increasingly biased toward the reach to A for longer delays.

In the account for visual habituation (Schöner and Thelen, 2006), the activation field spans visual features of the stimuli presented to the infants. While the infant is looking at a particular stimulus, localized input is provided to the field, inducing a peak of activation. The model postulates that such a perceptual peak stabilizes fixation of the stimulus. The model accounts for habituation by the build-up of a memory trace in the inhibitory layer of the perceptual field. Across trials, inhibition is strengthened, weakening the perceptual representation, and thus its stabilizing influence on fixation. The modeled infant will tend to look away from the stimulus to which it has habituated. Perone and Spencer (2013b) provide a elaborated neural dynamic account of visual habituation, in which the perceptual activation layer drives a working memory for the percept. As perceptual activation is strengthened by a memory trace, working memory passes a threshold. It is this new working memory for the percept that induces inhibition through its inhibitory layer that accounts for the weakening of the perceptual representation over viewing time and predicts looking away. That neural dynamic account of habituation may be seen as consistent with the Sokolov perspective (Sokolov, 1963) and its modern neural network implementation (Sirois and Mareschal, 2004), in which attention to a stimulus is stabilized while perceptual representations are being built, and destabilized thereafter.

In the neural dynamic models, perseveration in the reaching tasks and habituation in perceptual tasks are both caused by the build-up of activation through memory traces, but in different layers: Perseveration results from strengthened activation in an excitatory layer that drive motor behavior. Habituation results from strengthened activation in an inhibitory layer that weakens motor behavior. A unified account would postulate that, generically, memory traces strengthen activation both in excitatory and inhibitory layers. In such a unified account, familiarity preference in perceptual tasks and perseverative reaching in motor tasks originates from the memory trace in the excitatory layer. Habituation originates from the memory trace in the inhibitory layers. The unified account would be valid if habituation was also observed in motor tasks, so that a particular motor behavior becomes less likely when it is being performed repeatedly. Such motor habituation predicts a form of novelty preference, in which a habituated infant would then prefer to perform a new motor behavior over a familiar motor behavior.

Observations by Marcovitch et al. (2002) and Marcovitch and Zelazo (2006) in the A-not-B paradigm are consistent with this suggestion. These studies looked at how the number of reaches to A matters. In the experimental procedure the toy was hidden at the A location for one, six, or eleven trials before switching to the B location. This led to a U-shaped effect: Infants assigned to the single A trial condition did not perseverate at all. Infants in the traditional 6 A trial condition perseverated. Infants in the 11 A-trial condition were less likely to perseverate. The neural dynamic model of perseveration explains the absence of perseveration in the single A trial condition by the limited experience reaching to A, so that only a weak memory trace has been built. The model does not explain the reduced level of perseveration in the 11 A trial condition. A unified model would account for this reduction by the built up of an inhibitory memory trace that reflects habituation of the A reach.

In this article, we report an experiment that employs the experimental procedure of the habituation paradigm in a movement task. The experimental results provide evidence for habituation of movement generation that is specific to the direction of the movement: When the movement direction changes, we observe dishabituation. Moreover, we find a motor variant of Spencer-Thompson dishabituation. We then introduce a neural dynamic model that unifies previous accounts for habituation (Schöner and Thelen, 2006; Perone and Spencer, 2013b) and perseveration (Thelen et al., 2001; Dineva and Schöner, 2018). We use the model to account for the experimental finding. Finally, we extrapolate the model to a paradigm that involves motor selection in which the model accounts for perseverative reaching in the A-not-B paradigm (Smith et al., 1999) and the reduction of perseveration with increasing experience of an initial choice (Marcovitch et al., 2002).

## 2. Motor Habituation Experiment

**Figure 1:**
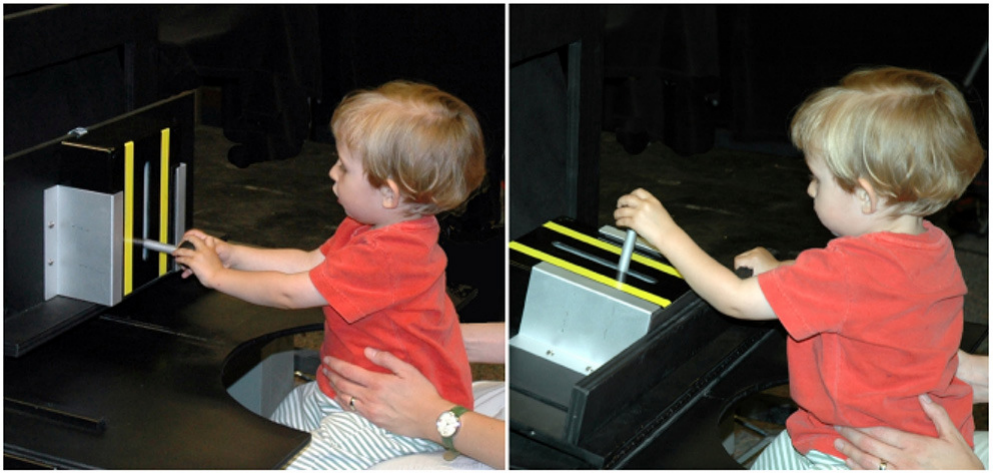
Experimental setting: A box with a lever was mounted on a black board with aluminum braces. The box could be oriented to enable vertical or horizontal movement of the lever. The box and environment were visually nondescript, besides two yellow stripes indicating the movement direction. While the lever was being moved, the box played music.

The motor habituation experiment mimicked the visual habituation paradigm. A box with a lever was repeatedly presented to toddlers (see Figure 1). Depending on how the box was presented, moving the lever entailed vertical or horizontal movements of the hand. Moving the lever lead to the box playing music and was, therefore analogous to fixating a stimulus in the visual habituation paradigm, which leads to visual stimulation. Only one movement direction was possible at a given time, the box's orientation was altered between habituation and test trials to probe for dishabituation and Spencer-Thompson dishabituation. Analogous to the A-not-B paradigm, action was elicited by pushing the box into the reaching space of toddlers and action was terminated by pulling the box out of reach when a trial ends.

### 2.1. Method

#### 2.1.1. Participants

Thirty eight 12-month-olds (23 boys, 16 girls) and 38 15-month-olds (22 boys, 17 girls) toddlers participated. Twenty one other toddlers were recruited but did not finish the experiment due to fussiness or technical problems. Their data were not included in the analysis. Toddlers of each age group were randomly assigned to two experimental conditions (starting with horizontal/vertical movement), resulting in 19 toddlers in each condition and age group.

#### 2.1.2. Apparatus and Data Acquisition

A lever mounted in the center of a box could be slid along a notch with a maximal range of motion of 11cm (Figure 1). To minimize visual distraction and the influence of perceptual habituation, the box was deliberately made visually boring, painted black with two yellow stripes parallel to the notch indicating the movement direction. We don't expect toddlers to habituate to such boring visual stimuli. Through a Labview data acquisition program, a computer recorded the moments in time when the lever was being moved and its current displacement. Based on the movement data, the computer controlled the speaker in the box, playing a sound file (Vivaldis piccolo concerto in c major) whenever the lever was being moved and turning it off when the movement stopped.

The box was placed on a board whose tilt angle relative to the table on which it was mounted could be adjusted to set the movement direction of the lever to horizontal or to vertical. The board could also be moved by the experimenter along a track closer or further away from the toddler. A semicircular notch cut out on the front of the table enabled the toddler to comfortably sit on a parent's lap facing the table and the box (see Figure 1). The parent sat on a rolling chair and positioned the toddler close to the edge of the table. The experimenter sat cross the table from the toddler, hidden by a curtain to reduce distraction.

During the experiment, the displacement of the lever was displayed on a screen in real time and an LED indicated whether the lever was being moved. The current trial number and elapsed time on the current trial were displayed and updated online. The total moving time and a habituation criterion were calculated online and used to control the timing of the experiment. The total moving time on the first three trials and the last three trials were displayed together with their ratio in percent. When the total moving time on the last three habituation trials fell below 50% of the total moving time on the first three habituation trials, an LED labeled “Reached Criterion” flashed. The experimenter then stopped the habituation phase by withdrawing the box, changed the angle of the box, and began the test trials.

The entire experimental session was videotaped for later review with two video cameras mounted in front of and on the right side of the toddlers, respectively.

### 2.2. Procedure

In the horizontal condition, toddlers of both age groups were first habituated to the horizontal movement direction, tested with the vertical movement direction on the first two test trials, and then tested again with the horizontal movement direction in two additional test trials. In the vertical condition, the same sequence was run through with horizontal and vertical movement direction swapped. Each trial lasted 15s. The toddler-controlled habituation criterion determined the end of the habituation phase, when the total moving time on the last three habituation trials fell below 50% of the total moving time on the first three habituation trials. This way, we apply the classic habituation criterion widely used in visual habituation to a motor task (see Colombo and Mitchell, 1990 for an overview of paradigms/criteria). The test phase started when the habituation criterion was met or the toddler finished 15 habituation trials.

Two experimenters were needed to run the experiment. Experimenter 1 operated the computer and informed experimenter 2 when a trial terminated. Experimenter 2 hid behind the curtain, withdrew and retrieved the box between trials, and changed the tilt angle of the board at the transition from the habituation to the test phase, and from test trial 2 to test trial 3. Experimenter 2 made the inter-trial interval constant through practice, which was about 11.5±1.5s across all trials, including the transition from one movement direction to the other.

After the parent completed the consent documents and the toddler was comfortable in the lab, parent and toddler sat down in front of the box. The toddler was given a short period of time to get familiar with the box before data collection started. The movement direction of the lever during this warm-up phase was the same as that in the following habituation phase. It followed a strict routine: The parents demonstrated the movement twice, held the toddlers hands on the handle twice, and then encouraged the toddlers to move the lever themselves. After the toddlers moved the lever independently for three times, the box was pulled back and pushed into place again to start the experimental trials.

At the beginning of each trial, the parents drew the toddlers attention to the box and put their hands on the lever if the toddlers did not voluntarily do so. Experimenter 1 started the 15s trial in the Labview program once the toddlers hands were on the knob of the lever. During a trial, the parents were asked not to interact in any way that would influence or distract the toddlers. However, they were allowed to say encouraging words when the toddlers moved the lever.

After the habituation criterion was met or the maximum of 15 habituation trials was exceeded, the orientation of the box was changed and two test trials started. Toddlers watched the experimenter rotating the box. No warm-up was given for the novel movement direction, the test trials started immediately after the last habituation trial. After two test trials in the new movement direction, the box was changed back to the familiar direction for two additional test trials.

### 2.3. Data Analysis

#### 2.3.1. Habituation and Dishabituation

Toddlers started and stopped moving the lever several times within a trial. The movement times of those episodes were summed for each trial. The habituation criterion was defined in terms of summed movement time as described above. Only 11 of 76 toddlers did not reach the habituation criterion so that their habituation phase ended after 15 trials. Dishabituation and Spencer-Thompson dishabituation were assessed through *t*-tests that compared the movement times in the test trials with the movement times of the last habituation trial for each age group and habituation condition.

As a second measure, the movement paths of all episodes within a trial were summed. Habituation manifests itself in movement path as well, when the habituation criterion defined on the basis of movement path is satisfied for the movement path in the last habituation trials. Dishabituation and Spencer-Thompson dishabituation were assessed based on movement path for each age group and habituation condition by *t*-tests.

#### 2.3.2. Handedness

Some toddlers switched hands across trials during the experiment. The hand toddlers used in each trial was coded from the video tape as left hand, right hand, or both hands. There were more hand switches in early trials than in late trials. Out of 76 participants, 26 switched hands during the experimental procedure (14 12-month-old, 12 15-month-old).

The decrease in movement time across subsequent trials with and without a hand switch was analyzed for those toddlers who switched hands. Only trials during the habituation phase were considered. The decrease of movement time on early trials (first three habituation trials) was compared to the decrease of movement time on late trials (last three habituation trials) for trials with and without a hand switch and for all age groups and conditions in an ANOVA.

### 2.4. Results

#### 2.4.1. Habituation and Dishabituation

**Figure 2:**
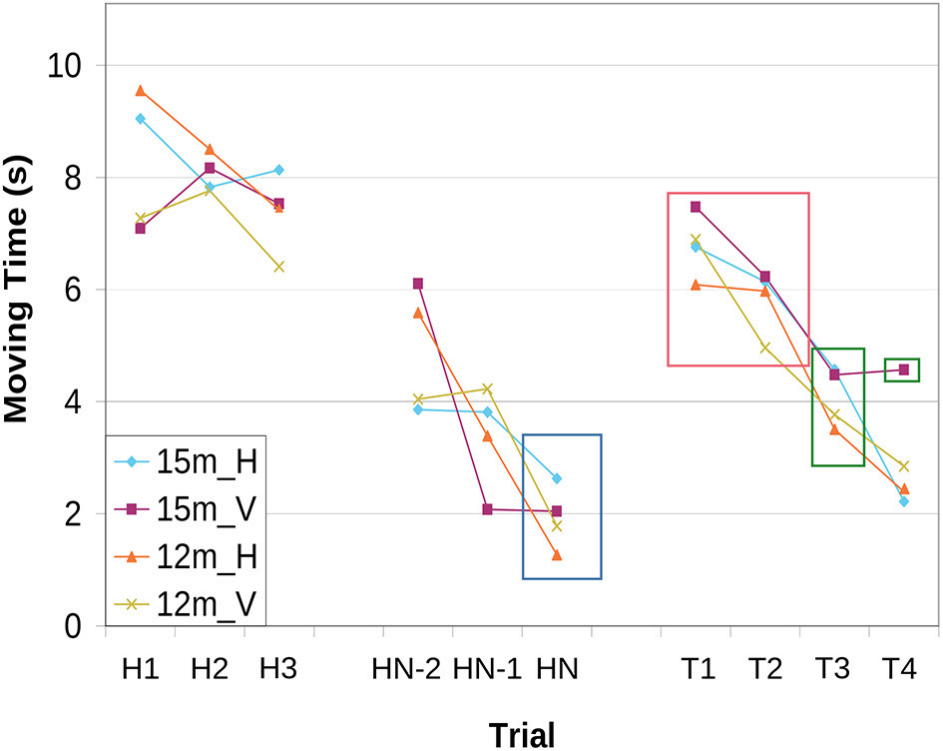
Experimental results: Average movement times for the first (H1-H3) and last three habituation trials (HN-2, HN-1, HN), as well as the test trials (T1-T4). Movement times are averaged across age group (12 or 15 months) and habituation condition, horizontal (H) or vertical (V). Average movement time satisfies the habituation condition in all groups in the last habituation trial, HN, marked by the blue box. The orange box marks significant dishabituation (difference to last habituation trial, HN) to the new movement direction in T1 and T2. Spencer-Thompson dishabituation is significant (difference to HN) in some trials and for some age groups/conditions, marked by the green boxes.

Figure 2 shows the average movement times on the first three and last three habituation trials and on the test trials for each age group and condition. Average movement times decrease during the habituation phase, satisfying the habituation criterion in all age groups and conditions. When the new movement direction is tested, average movement times increase compared to the last habituation trial. This provides evidence for dishabituation, which is significant, at *p*-value < 0.05, in all age groups and conditions in the first and second test trial (see orange box in Figure 2). When the original movement direction is tested again in test trials three and four, movement time increases sightly compared to the last habituation trial. This provides evidence for Spencer-Thompson dishabituation, which is significant for all age groups and conditions on test trial three. On test trial four, it is significant only for 15-month-old in the vertical habituation condition (see green boxes in Figure 2).

Results based on the second measure of movement, the summed movement path per trial, have the same structure: The habituation criterion is met on the last habituation trial in all groups/conditions. Average movement paths lengthened on test trials one and two. This dishabituation to the new movement direction was significant in all age groups and conditions. In test trials three and four, average movement paths lengthened slightly compared to the last habituation trial. Spencer-Thompson dishabituation was significant in T3 for all age groups and conditions, in T4 only for 15-month-old in the vertical movement direction.

These results provide evidence for habituation to movements. The observed dishabituation shows that habituation is specific to a specific movement direction, suggesting the existence of novelty preference in motor behavior.

#### 2.4.2. Handedness

**Figure 3:**
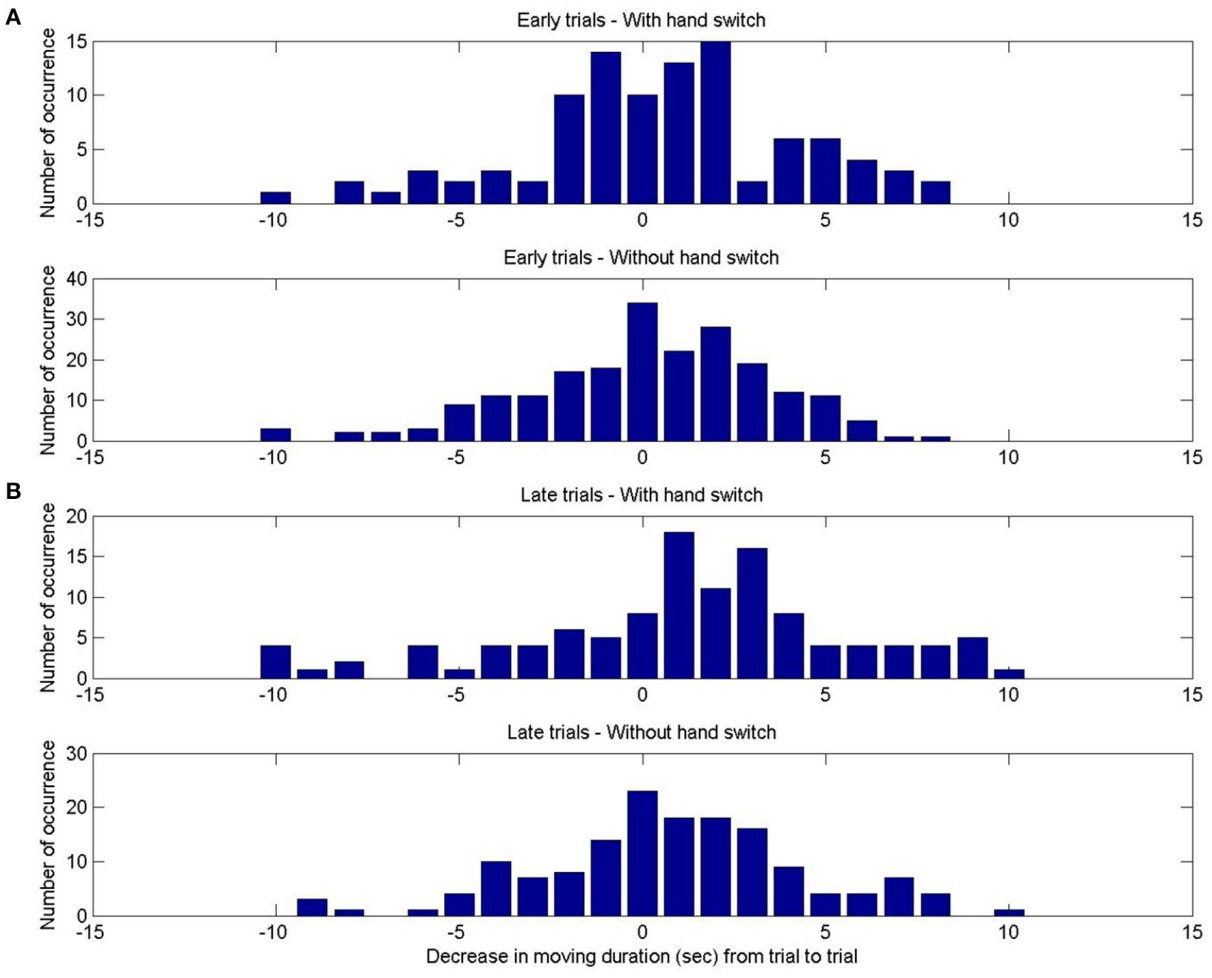
Experimental results: Distribution of decrease in movement time between two trials. **(A)** In the first three habituation trials, when a hand switch occurred between trials (top) and without hand switch (bottom). **(B)** In the last three habituation trials, when a hand switch occurred between trials (top) and without hand switch (bottom).

The only significant main effect on change of movement time across subsequent trials reflected that movement time decreases more strongly in late trials than in early trials. The decrease in movement time did not interact with age or condition, nor does it interact with the presence or absence of a hand switch. Figure 3 shows the distribution of movement time decreases from one trial to the next when a switch of hand occurred as contrasted to movement time decreases from one trial to the next when no switch of hand occurred. These distributions are shown separately for trials early and late during habituation.

The result suggests that habituation is not specific to the effector used. Such a dependence would predict less decrease or an increase of movement time after a hand switch. This is consistent with ascribing habituation to a level higher than the effector specific movement generator, for example, to a level representing an intention to move the lever. This informs the choice of level of description in the model.

## 3. Neural Dynamic Model of Motor Habituation

### 3.1. Motor Habituation Model

**Figure 4:**
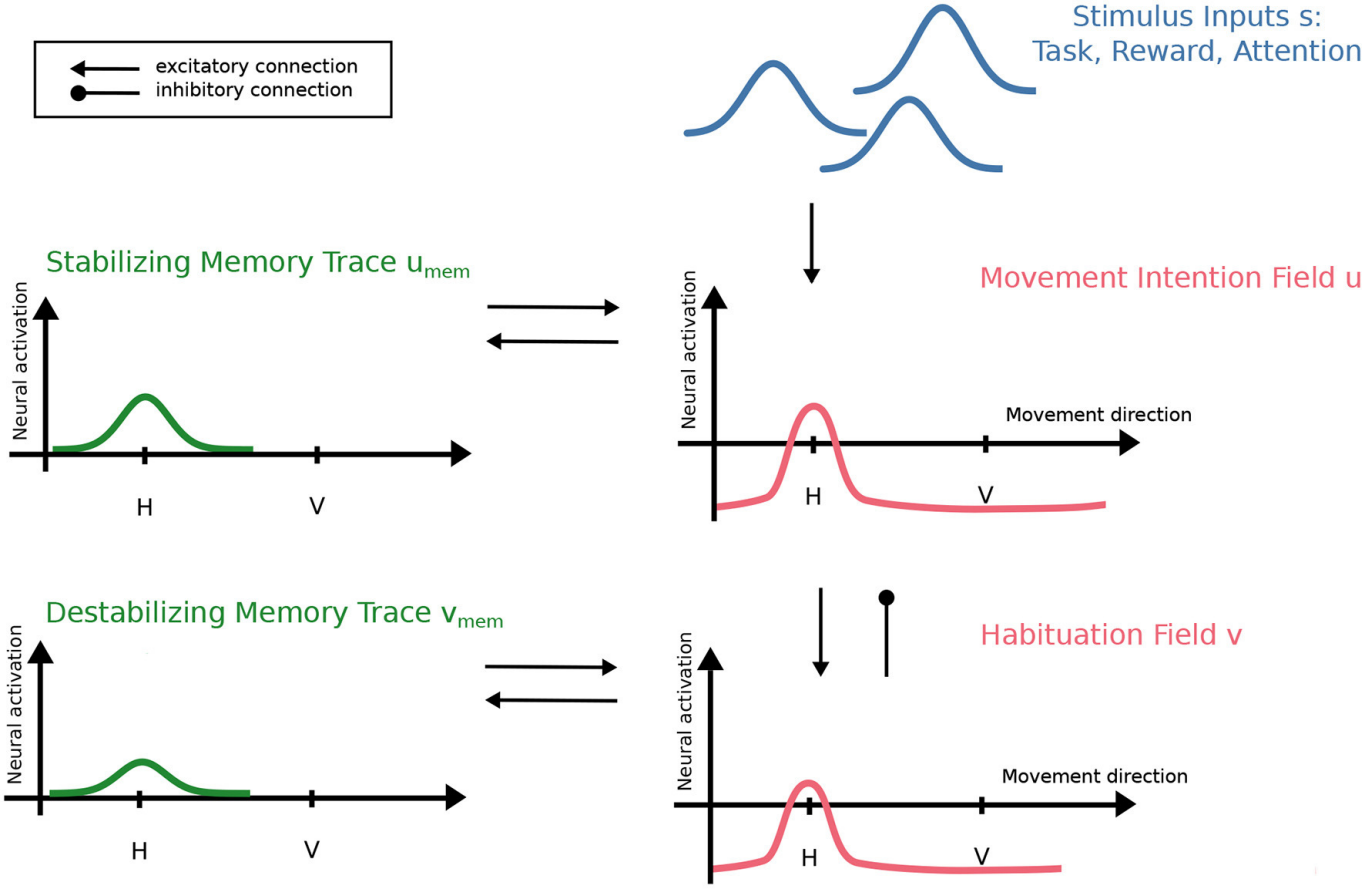
DFT model of motor habituation. The intention field, *u*, receives stimulus inputs, *s*, that models the visual perception of the box and lever, the perception of rewarding outcomes, or stimulation by a parent. The intention field provides input to the habituation field, *v*, which in turn inhibits the intention field. Both fields are defined over the movement direction, *x*, sampled at horizontal (H) and vertical (V) movement directions in the experiment. A supra-threshold peak at a location, *x*, in the intention field indicates that a movement in that direction is being generated. Memory traces reflect the recent history of supra-threshold activation in both fields and provide input back to the fields. They facilitate peak formation and, thus, account for the stabilization and destabilization of movement intentions.

To account for motor habituation as observed in the reported experiment, we unify previous neural process accounts for perceptual habituation (Schöner and Thelen, 2006; Perone and Spencer, 2013b) and perseverative reaching in the A-not-B paradigm (Thelen et al., 2001; Dineva and Schöner, 2018) that were based on the framework of Dynamic Field Theory (DFT) (Schöner et al., 2016). A two-layer neural dynamic field is defined over movement direction *x* (see Figure 4). At the first layer, an excitatory field, *u*(*x, t*), represents the intention to move in a particular direction, *x*. At the second layer, an inhibitory field, *v*(*x, t*), mediates habituation. It receives excitatory input from the intention field, *u*(*x, t*), which it in turn inhibits. Activation in both fields evolves continuously in time as described by neural dynamics (described in mathematical detail below, see Equation 1 in section 3.1.1).

The intention field evolves under the influence of a variety of inputs, *s*(*x, t*), that reflect perceptual information (see below). Recurrent connectivity within the fields contributes more strongly than such external inputs, however. Local excitatory connectivity within the intention field stabilizes localized patterns of activation against decay. Input from the inhibitory field stabilizes peaks against diffusive spread, but may also weaken activation patterns in the intention field. Only field locations that are sufficiently activated engage in neural interaction, as modeled by a sigmoid threshold function that makes the neural dynamics nonlinear (see Equation 4).

Without input, activation in both fields is at a negative resting level. When input pushes activation at some field location through the threshold of the sigmoid, the sub-threshold pattern of activation becomes unstable. Driven by local excitatory interaction, activation evolves to a supra-threshold stable state, that is, a localized peak of activation. In the excitatory field, this represents the intention to move the hand in a particular direction that is encoded by the location of the peak along the field dimension^1^.

Various perceptual inputs to the intention field model the experimental procedure. Task input, *s*_T_, represents that a box affording a particular movement direction is within reach. The trials and inter-trial intervals are modeled by varying task input in time. Reward input, *s*_R_, models the strengthening of an active movement intention when the rewarding outcome, the music, is perceived. In the simulations, this input is only present while a supra-threshold peak exists that would induce lever movement in the (unmodeled) motor system. A third input models the parent's action of drawing attention to the box and encouraging the child to move the lever. This attention input, *s*_A_, is applied while task input is provided to the intention field but no supra-threshold peak has yet formed. It may be strong enough to push the intention field through the detection instability.

A peak in the intention field decays when the supra-threshold state becomes unstable so that activation falls back to a sub-threshold state. This happens in the reverse detection instability at lower levels of input than the detection instability. The decay of a peak reflects the decision to stop moving the lever. This happens when the task input is removed at the end of a trial or when inhibitory input from the habituation field becomes sufficiently strong.

Habituation (and perseveration) reflect the history of activation. The model represents that history through memory traces of both activation layers, *u* and *v*, of the model. In DFT, dynamic memory traces model a simple form of learning (akin to the dynamics of the bias inputs in connectionist networks). The memory trace builds on a slower time scale at locations with supra-threshold activation (see Equation 5) and decays if those locations fall below threshold while supra-threshold activation is present anywhere else in a field. Without supra-threshold activation in a field, the memory trace remains constant. Memory traces act like a locally enhanced resting level, preshaping the activation patterns in the field and facilitating peak formation at these locations. The memory trace, *u*_mem_, of the intention field thus accounts for the stabilization of movement intentions. The memory trace *v*_mem_, of the habituation field accounts for the destablization of movement intentions by enhancing inhibition.

When there are localized inputs at multiple field locations, only one peak may form in the intention field due to inhibitory input from the habituation field. The motor habituation experiment does not probe such selection decision as only a single movement direction is afforded at any moment in time. We will examine situations involving selection in the model, to connect the account to models of motor decision. Memory traces in excitatory fields of such models have previously been used to account for pre-trial effects (Erlhagen and Schöner, 2002; Dineva and Schöner, 2018), and perseveration (Thelen et al., 2001).

In DFT models of visual habituation, activation in the excitatory perception field is defined over features of the visual percept (Schöner and Thelen, 2006; Perone and Spencer, 2013b). This perceptual activation is assumed to stabilize fixation. Reduced activation due to the build-up of inhibition then promotes looking away, a signature of habituation (Schöner and Thelen, 2006; Perone and Ambrose, 2016), and preferential looking (Goldberg and Schöner, 2007; Perone and Spencer, 2013a,b).

#### 3.1.1. Mathematical Formulation

The evolution of the intention and habituation fields is modeled by this neural dynamics:


$$\begin{array}{l} \tau_{\text{u}}\dot{u}(x,t)=-u(x,t)+h_{\text{u}}+s(x,t)+\int k_{\text{uu}}(x-x^{\prime })g(u(x^{\prime },t))\text{d}x^{\prime }\\ \;-{\int \;}^{\text{​}}k_{uv}(x-x^{\prime })g(v(x^{\prime },t))dx^{\prime }\\ \;+\;\int^{\text{​}}\;k_{uu_{\text{mem}}}(x-x^{\prime })g(u_{\text{mem}}(x^{\prime },t))dx^{\prime }+\tau_{v}q\xi_{v}(x,t),\;(1)\\ \;\tau_{v}\dot{v}=-v(x,t)+h_{\text{v}}+\int k_{\text{vu}}(x-x^{\prime })g(u(x^{\prime },t))\text{d}x^{\prime }\\ \;+\int k_{{\text{vv}}_{\text{mem}}}(x-x^{\prime })g(v_{\text{mem}}(x^{\prime },t))\text{d}x^{\prime }+\tau_{\text{v}}q\xi_{\text{v}}(x,t). \end{array}$$

Independent Gaussian white noise, ξ_*i*_(*x, t*), with strength *q* is applied to all field locations. The time scales, τ_*i*_, determine how fast activation in the fields evolves. Without inputs, activation in the fields is at the negative resting level *h*_*i*_ < 0. The input, *s*(*t, x*), sums over the three sources of stimulation and is applied to the intention field *u* during the experimental procedure. Stimulus components, *s*_*k*_(*x*), are modeled as Gaussian functions:


(2) $$s_{k}(x)=\frac{a_{k}}{\sqrt{2\pi }\sigma_{\text{exc}}}\exp\left\{-\frac{{\left(x-x_{0}\right)}^{2}}{2\sigma_{\text{exc}}^{2}}\right\},$$

with width σ_exc_ and amplitude *a*_*k*_. The index *k*= T,R,A corresponds to the Task, Reward, or Attention input. The Gaussian functions are centered on *x*_0_ = *H* or *x*_0_ = *V* for a horizontal or vertical movement direction, respectively.

Lateral interactions within and between the fields are determined by interaction kernels, *k*_*ij*_


(3) $$k_{ij}(x-x^{\prime })=\frac{c_{ij}}{\sqrt{2\pi }\sigma_{ij}}\exp\left\{-\frac{{\left(x-x^{\prime }\right)}^{2}}{2\sigma_{ij}^{2}}\right\}+c_{ij,\text{glob}},$$

where the first index corresponds to the target field and the second to the source field of the projection. The Gaussian part models local interaction within a field (*i* = *j*) or coupling to other fields (from field *j* to field *i*) with width σ_*ij*_ and strength *c*_*ij*_. Global interaction is determined by the constant *c*_*ij*, glob_ which is applied to all field locations.

Only field locations that have sufficient levels of activation engage in lateral interaction. The output of a field *u* is determined by a sigmoid function with threshold at zero, whose steepness is given by β:


(4) $$\begin{array}{r} g\left(u\right)=\frac{1}{1+\exp\left(-\beta u\right)}. \end{array}$$

The memory trace of the intention field grows with the time scale τ_build_ more slowly than the fields:


(5) $$\begin{array}{l} {\dot{u}}_{\text{mem}}(x,t)=\tau_{\text{build}}^{-1}\left[-u_{\text{mem}}(x,t)+g(u(x,t))\right]g(u(x,t))-\tau_{\text{decay}}^{-1}u_{\text{mem}}(x,t)\left[1-g(u(x,t))\right],\\ \;-\tau_{\text{decay}}^{-1}u_{\text{mem}}(x,t)\left[1-g(u(x,t))\right], \end{array}$$

as long as there is supra-threshold activation at any location in the corresponding field. Otherwise, the memory trace remains constant (${\overset{∙}{u}}_{\text{mem}}=0$). Activation in the memory trace thus decays competitively only when there is supra-threshold activation at other field locations. In general, the time scale for decay, τ_decay_, is slower than for building the memory trace. The dynamics of the memory trace, *v*_mem_, of the habituation field is described by the same dynamics, although the time scales may differ.

#### 3.1.2. Constraints on Model Parameters

The experimental procedure, observations during the experiment, and qualitative assumptions about the results provide constraints for setting many of the parameter values of the model:

(1) Toddlers moved the lever only after the warm-up phase during which they were encouraged by their parent. We assume this to be a critical part of the procedure that enabled the toddlers to associate the lever moving action with the rewarding outcome, the music. We expect that they would not be interested to move the lever without the music. The amplitude of the task input, *s*_T_, is chosen, therefore, such that task input alone is not sufficient to elicit a supra-threshold peak in the intention field. Only task input in combination with input from the stabilizing memory trace or the attention input induces supra-threshold activation in the intention field.

(2) Since toddlers do not try to move the lever while the box is out of reach, input from the stabilizing memory trace, *u*_mem_, to the intention field alone is assumed to be insufficient to induce a peak. This constrains the coupling strength, *c*_u_u__mem__, to be less than the absolute value of the intention field's resting level |*h*_u_|. A combination of at least two of the three sources of inputs, task input, attention input, and input from the excitatory memory trace is assumed necessary to induce a detection instability in the intention field.

Since the rewarding input is only applied when there already is supra-threshold activation in the intention field, it does not play a role in inducing a detection instability. However, it further stabilizes the decision when the attention input is removed. This models that toddlers who were encouraged to move the lever at the beginning of a trial kept moving when perceiving the rewarding music without a need for continued stimulation from their parent.

(3) Typically, after a few trials toddlers stopped moving the lever even while the box was within reach. The coupling strength, *c*_uv_, from the habituation to the intention field is thus chosen such that the inhibitory input to the intention field becomes larger than the sum of task input and input from the memory trace, *u*_mem_. This makes it possible that a supra-threshold peak in the intention field can be destabilized by inhibition from the habituation field.

At the beginning of a trial, the parent encourages his or her child to move the lever. The coupling strength, *c*_uv_, is thus assumed to be smaller than the attention input combined with the task input and input from the stabilizing memory trace so that the attention input may elicit a peak in the intention field despite strong inhibition from the habituation field.

(4) Since there is no self-excitation within the inhibitory layer, the coupling strength, *c*_vu_, must be strong enough for the intention field to cause supra-threshold activation in the habituation field.

(5) To model Spencer-Thompson dishabituation, the destabilizing memory trace of the habituation field, *v*_mem_, must decay faster than that of the intention field. Thus, after a new movement was performed there is less inhibition at the field location to which the model was habituated.

Supra-threshold activation at another location of the habituation field is necessary for the memory trace, *v*_mem_, to decay at an initial location. To obtain Spencer-Thompson dishabituation, a stimulus that is sufficiently different from the initial stimulus must thus be presented after habituation. This constrains the metric overlap between field locations and the respective widths of projection kernels.

(6) The stabilizing memory trace of the intention field, *u*_mem_, must grow faster than the destabilizing memory trace of the habituation field, so that it is predominant in early trials. The coupling strength from the habituation field to the intention, *c*_uv_, must be stronger than its coupling to the stabilizing memory trace, so that habituation prevails in later trials. This cannot be deduced directly from the motor habituation data, but is consistent with the pattern of early familiarity and a late novelty preference found across a variety of selective tasks.

(7) The experimental results show that the response to the new movement direction is stronger on the first test trial than for the old movement direction on the last habituation trial, but typically not as strong as on the first habituation trials. This points to the existence of global component of habituation across movement directions. Thus, the projection kernel from the memory trace *v*_mem_ to the habituation field is assumed broader than the projection kernel from *u*_mem_ to the intention field, including a global (=constant) component.

Table 1 provides an overview of the set parameter values.

**Table 1:** Parameter values of the habituation model.

**Parameter**	**Value [a.u.]**	**Meaning/Constraints**
β	6	Steepness of sigmoid function
τ_u_	40	Time scale of *u*
*h*_u_	−1.2	Resting level, |*h*_u_|≥*s*_T_
*c*_uu_	1.2	Local excitation in *u*, stabilizes peak decisions in *u*
σ_uu_	2.5	Width of excitatory kernel, σ_ij_ ≪ field size for distinct peaks
*c*_u_u__mem__	0.8	Local input from memory trace, facilitates peak formation at familiar locations
σ_u_u__mem__	2.5	Width of excitatory kernel
*c*_u_u__mem_, glob_	0.2	Global input from memory trace
		*c*_u_u__mem__+*c*_u_u__mem_, glob_ ≤ |*h*_u_| → no spontaneous movement without stimulus inputs
*c*_uv_	−1.8	Local inhibition from *v*, leads to habituation
σ_uv_	5	Width of inhibitory kernel, broader than excitatory kernel
*c*_uv, glob_	−0.4	Global inhibition from *v*, for habituation and selection decisions
		|*c*_uv_|+|*c*_uv, glob_|≥*s*_T_+*c*_u_u__mem__+*c*_u_u__mem_, glob_ for “full” habituation
τ_v_	2	Time scale of *v*, fast inhibition for global inhibition τ_v_≪τ_u_
*h*_v_	−1.2	Resting level, |*h*_v_| ≤ *c*_vu_ so that *u* drives supra-threshold activation in *v*
*c*_vv, glob_	−0.1	Global inhibition in *v*
*c*_vu_	2.5	Local excitation from *u*, drives activation in *v*
σ_vu_	2.5	Excitatory kernel width
*c*_v_v__mem__	3	Local excitation from *v*_mem_, modulates strength of habituation
σ_v_v__mem__	2.5	Excitatory kernel width
*c*_v_v__mem_, glob_	0.35	Global excitation from *v*_mem_, modulates strength of habituation and Spencer-Thompson dishabituation
τ_u_mem_, build_	200	Building time scale of stabilizing memory trace
		τ_u_mem_, build_≫τ_u_
τ_u_mem_, decay_	2,000	Decaying time scale of stabilizing memory trace
τ_v_mem_, build_	600	Building time scale of destabilizing memory trace
		τ_v_mem_, build_≫τ_u_mem_, build_ for familiarity preference
τ_v_mem_, decay_	1,000	Decaying time scale of destabilizing memory trace
		τ_v_mem_, decay_ ≤ τ_u_mem_, decay_ for Spencer-Thompson dishabituation
*s*_T_	1.0	Task input
*s*_R_	1.0	Reward input
*s*_A_	1.5	Attention input

*Parameters not shown were set to zero in the simulation. The third column addresses the meaning of parameters in the model or constraints we defined for a parameter. For a detailed analysis of parameter constraints see section 3.1.2*.

### 3.2. Simulations

For numerical simulation, the model was implemented in MATLAB using the toolbox COSIVINA for dynamic field architectures^2^. The simulation emulated the procedure of the motor habituation experiment. In the habituation phase, the Gaussian task input, *s*_T_, is repeatedly applied to the intention field at location representing horizontal movement, indicating both that the box is in reach and affords a horizontal movement direction. Attention input is added to the same field location when activation does not reach supra-threshold activation within 5s. On the first trial it is not possible to induce a peak in the intention field because there is no input yet from the stabilizing memory trace *u*_mem_. This is when attention input is applied simultaneously with task input, pushing the intention field through the detection instability. This accounts for the warm-up phase of the experiment.

Once a peak forms in the intention field or the attention input is applied, the trial starts. Task input is maintained for another 15s from that moment on. In the experiment a trial started as soon as the toddlers had their hands on the lever and lasted from then on 15s. The reward input is added as soon as activation in the intention field reaches the threshold. Any attention input is then removed. At the end of the trial all stimulus inputs are removed for an inter-trial period of 12s before the task input is applied again at the same field location. With activation reaching the threshold or the attention input added, a new trial begins.

On each trial, the number of time steps at which supra-threshold activation is observed in the intention field are accumulated as a measure for movement time. The simulated movement time is based on the intention to move alone (neglecting to model actual movement generation). As in experiment, the habituation phase ends when the habituation criterion is met, that is, the movement time of the just previous three trials is less than 50% of the movement time of the first three trials, or after a maximum of 15 trials.

In the subsequent test phase, the task input is applied twice at the new, vertical field location, modeling that the box is in reach but was rotated. Then, task input is again applied twice at the original field location to probe Spencer-Thompson dishabituation. The trial and inter-trial periods as well as the conditions for applying the attention and reward input remain unchanged.

### 3.3. Results

#### 3.3.1. Simulation Results in the Habituation Paradigm

**Figure 5:**
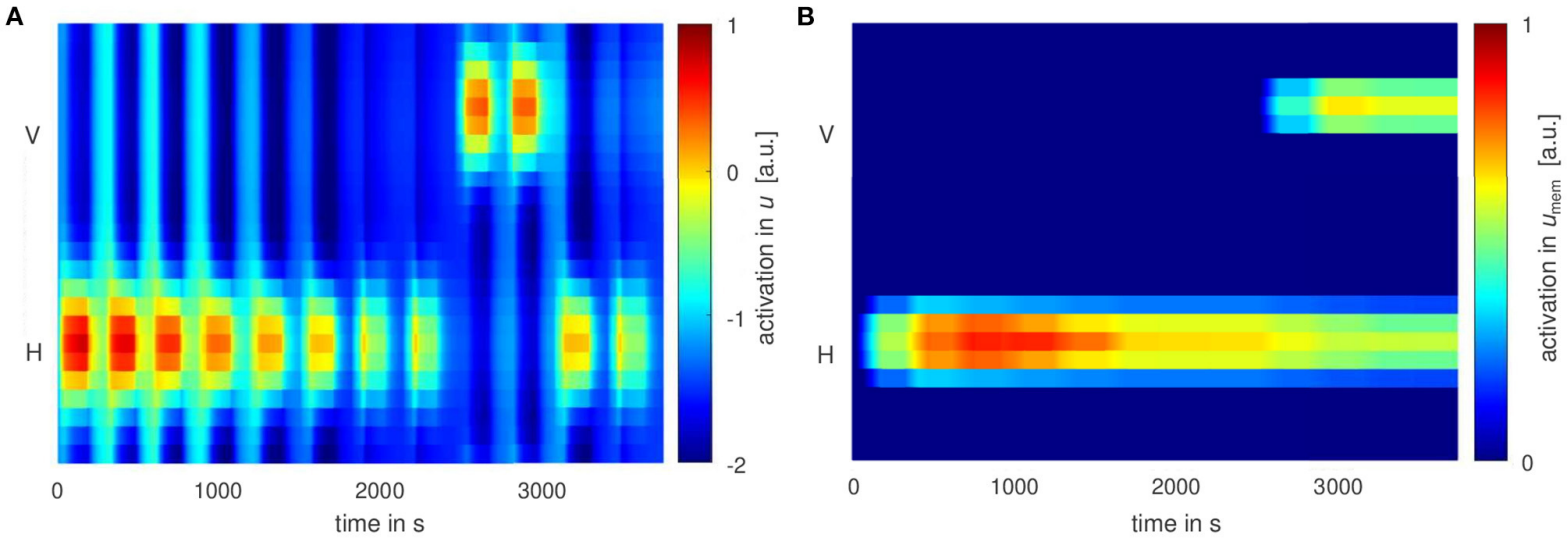
**(A)** Evolution of activation in the intention field *u*. Supra-threshold activation (orange-red color) is caused by stimulus inputs applied to the respective field locations repeatedly. **(B)** Evolution of the corresponding memory trace *u*_mem_. The memory trace grows at supra-threshold field locations while it decays at all other locations.

Figure 5 shows an exemplary time course of activation in the movement intention field *u* as well as its memory trace *u*_mem_. Time courses of different simulation runs vary due to noise in the fields. Field parameters remained the same in all simulations. Once task and attention input (not shown in Figure 5) are applied at field locations representing a horizontal or vertical movement direction, activation at those locations becomes supra-threshold (orange-red color in Figure 5A). Supra-threshold activation in *u* corresponds to the intention to move the lever. Between trials, when no inputs are applied to *u*, activation in the movement intention field remains sub-threshold (green-blue color). This corresponds to the observation that toddlers did not try to move the lever when the box was out of reach.

During the habituation phase, activation at horizontal field location becomes supra-threshold repeatedly. Over time, amplitude and time-duration of such peaks decrease (areas of red-orange color are narrower than in the first trials in Figure 5A) because of increasing inhibition from the habituation field (not shown in Figure 5). We assume that supra-threshold activation in the movement intention field *u* leads to movement generation, that is moving the lever in horizontal direction. Thus, the time of supra-threshold activation in *u* correlates with movement time measured in the motor habituation experiment. We also expect the amplitude of supra-threshold activation to modify movement generation. It was observed that toddlers moved the lever in several moving episodes rather than moving it continuously during a trial. The amplitude may modify the length of such episodes or the moving speed during an episode.

After the habituation criterion was met, input is applied to vertical field location. Amplitude and time of supra-threshold activation in *u* are reinstated (red-orange area is broader than in the previous trials) because inhibition from the habituation field is not as strong as at horizontal field location yet. This models dishabituation to a new movement direction. In the last two trials, horizontal task input is applied again and the intention field again becomes supra-threshold at horizontal field location. Inhibition from the habituation field is decreased compared to the last habituation trial which leads to increased movement time (red-orange area is broader than in the last habituation trial). In the experiment this is observed as Spencer-Thompson dishabituation.

Figure 5B shows how the stabilizing memory trace *u*_mem_ grows at locations of supra-threshold activation in the movement intention field *u*. The memory trace grows and decays slower than the field. When *u* becomes supra-threshold at the new, vertical field location, activation in *u*_mem_ grows at vertical field locations as well, while decaying at horizontal location. Input from *u*_mem_ to the movement intention field *u* facilitates peak formation in *u*. Activation in the habituation field *v* is driven by input from the movement intention field *u* and has a similar pattern as shown in Figure 5A. Its corresponding memory trace *v*_mem_ builds slower than the memory trace *u*_mem_ but decays faster.

**Figure 6:**
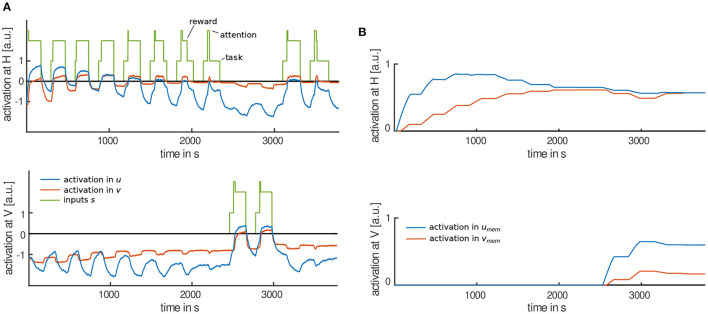
**(A)** Exemplary time courses of the movement intention *u* (blue), the habituation field *v* (red) and stimulus inputs applied (green) at the horizontal (top) and vertical (bottom) field location. **(B)** Corresponding time courses of the memory trace, *u*_mem_, of the intention field (blue) and the memory trace, *v*_mem_, of the habituation field (red) at horizontal (top) and vertical (bottom) field location.

For a detailed analysis of the model, Figure 6 shows a cut through the movement intention field at horizontal (top) and vertical (bottom) field locations as well as activation in the habituation field, stimulus inputs and corresponding memory traces (b). In the first habituation trial, task and attention input are applied to the movement intention field at horizontal movement direction (see Figure 6A, top). Once activation in *u* pierces the threshold of zero, attention input is omitted and reward input is applied. Activation stays above the threshold until task and reward input are removed at the end of the trial. Supra-threshold activation in the movement intention field drives growth of its memory trace (Figure 6B, top). This stabilizing memory trace provides input back to the movement intention field so that in the following trials it goes through the detection instability faster and without attention input being applied (trials 2–4 in Figure 6A, top). This predicts that toddlers would move the lever spontaneously on these trials.

When the movement intention field becomes supra-threshold input is passed to the inhibitory layer, the habituation field *v*. Responses in the habituation field are delayed compared to activation in the intention field because it is driven by the intention field only once activation there reaches threshold. Supra-threshold activation in the habituation field drives its memory trace *v*_mem_. This destabilizing memory trace *v*_mem_ provides input back to the habituation field and facilitates peak formation in the following trials, leading to a stronger inhibition of the movement intention field. So, levels of activation in the movement intention field decrease over trials. As a result, activation in the movement intention field does not go through the detection instability, when task input is provided in trials 5–8. Therefore, attention input is applied again. This predicts that toddlers would not move the lever spontaneously on these trials. As a results, parents would need to draw toddlers' attention to the lever.

With increasing inhibition from the habituation field, activation in the intention field is pushed below the threshold even before the trial ends (trials 6–8 in Figure 6A). This reproduces the observation in the experiment that toddlers stopped moving the lever although the box was still within reach. The reverse detection instability induced in the intention field is amplified by the removal of the reward input once activation falls below the threshold, making it less likely that the intention field goes through the detection instability a second time. The reward input may also amplify a detection instability, as it is applied once activation in the intention field reaches the threshold, which leads to even higher levels of activation in the intention field. When activation in the intention field goes through the reverse detection instability before a trial ends, movement time decreases. The habituation phase continues until the habituation criterion is met. In the simulation run shown in Figure 6, the criterion is met in the eighth trial.

In the first test phase, task input is applied at field locations representing vertical movement direction (see Figure 6A, bottom). Again, attention input is needed to push the intention field through the detection instability at the new field location as there is insufficient input yet from the stabilizing memory trace (trials 9 and 10, in Figure 6B). Here, the model lacks knowledge that toddlers might actually have about the box playing music even when in a new orientation. However, movement time is reinstated as soon as a peak forms in the intention field and remains until task input is removed. This is how the model accounts for dishabituation to a new movement direction.

In the second test phase, the task input is applied again at horizontal field location of the intention field (see Figure 6A, top). Once activation goes through the detection instability, with the help of attention input, it remains supra-threshold for a longer time period than in the last habituation trial. This is because the destabilizing memory trace of the habituation field decays faster than the stabilizing memory trace during the first test trials while task input was applied at the competing field location. Thus, there is less inhibition from the habituation field compared to the last habituation trial, while the impact of the stabilizing memory trace is about the same (see Figure 6B, top). In the last test trial, activation in the destabilizing memory trace has grown again and inhibition from the habituation field is strong enough to push activation in *u* through the reverse detection instability before the trial ends. The model thus accounts for Spencer-Thompson dishabituation in the third but not in the fourth test trial.

**Figure 7:**
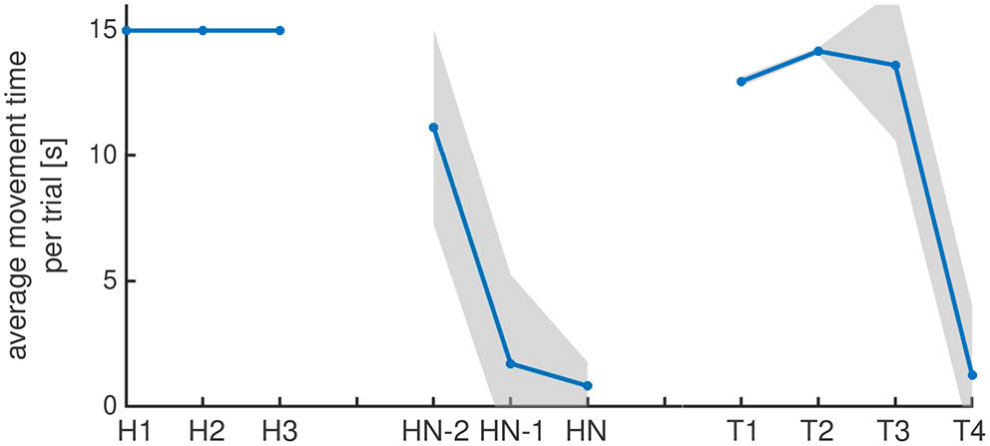
Movement times averaged across simulation runs that were aligned as in the experimental analysis: The first and the last three habituation trials, the test trials in vertical movement direction (T1 and T2) and the test trials in the original movement direction (T3 and T4). The standard deviation across simulation runs due to noise in the fields is shown in gray.

Figure 7 shows movement times from the model, averaged across 50 simulations runs (analogously to the experimental movement times in Figure 2). Because time courses of activation and thus movement times fluctuate across trials, the habituation criterion is met at different trial numbers in different simulation runs. In the first trials, movement time is saturated since activation in the movement intention field remains supra-threshold as long as the 15s trial lasts.

The model reproduces the reduction of average movement time on the last three trials of the habituation phase over to the average movement time in the first three trials. On the subsequent two test trials, the average movement time is reinstated, a signature of dishabituation. In the second test phase, average movement times are increased in the third test trial (T3) compared to the last habituation trial, a signature of Spencer-Thompson dishabituation. The model shows no significant Spencer-Thompson dishabituation in the fourth test trial.

As stable states, supra-threshold peaks in neural dynamic fields resist noise. Noise may have a strong effect on the system's state near an instability, however. In the model, reward input amplifies small fluctuations when the system is close to the (reverse) detection instability as noise drives activation to positive (or negative) levels. Due to the memory traces, the history of supra-threshold activation has a direct impact on the future time course of activation, leading to variance across simulation runs. [Analogous observations were reported in Perone and Spencer (2013b) in a model of preferential looking.] Figure 7 reflects this fact through the increase of the standard deviation of movement time increases over trials. In the first two test trials (T1, T2) standard deviation is decreased because activation in memory traces at horizontal field location affect activation at vertical field location only marginally. When task input is again provided at horizontal field location in test trials three and four, standard deviation increases.

#### 3.3.2. Discussion of the Habituation Results

The model simulations are qualitatively in agreement with the experimental data. The model accounts for habituation to a familiar movement direction by a reduced time of movement intention and for dishabituation to a new movement by restoring of movement time. In the third test trial the model also captures Spencer-Thompson dishabituation. We did not try to push quantitative fits beyond what is shown in the figures. The experiment provides evidence for habituation to a movement based on both movement time and movement path. The model operates at the level of movement intentions, so that movement time is accounted for as the time periods during which movements could be generated. Quantitative fits of movement time and path length would need to take processes underlying the actual generation of motor commands into account. These may contribute delays that by themselves depend on the level of activation at the intention level. So, while we expect movement intention to correlate with movement times observed in the experiment, an exact match is not expected. For instance, the modeled movement time is saturated in the first habituation trials (see Figure 7) corresponding to the intention or willingness to move throughout the whole trial. In the experiment, movement episodes rather than continuous movements throughout the trial were observed and we expect the actual moving time to be less than the modeled time. Figure 6A shows that the amplitude of activation is decreasing in the first habituation trials, probably affecting movement generation and leading to shorter movement episodes. Similarly, the variance induced at the level of movement intention is not necessarily comparable to variance observed at the level of actual movement generation. Moreover, different sources of variation beyond random stochastic perturbations may contribute to experimental assessments of variance, including individual differences (best modeled by differing parameter values), different age groups and variance at the level of sensory inputs.

#### 3.3.3. Testing the Effect of Outcome

**Figure 8:**
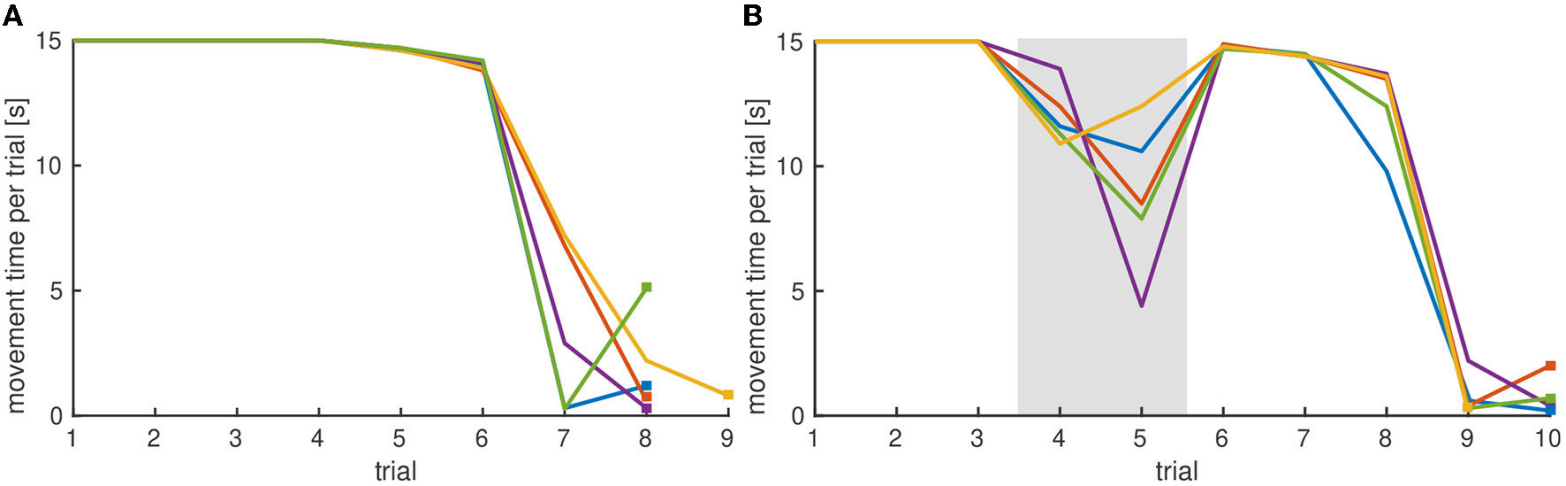
**(A)** Evolution of movement time over trials for five simulation runs with reward input applied in all trials. The habituation criterion is met at different trials for each run, indicated by the square marker. **(B)** Evolution of movement time over trials for five simulations for which reward input was omitted on trials 4 and 5, marked by the gray area. The habituation criterion is met at later trials, marked by a square.

**Figure 9:**
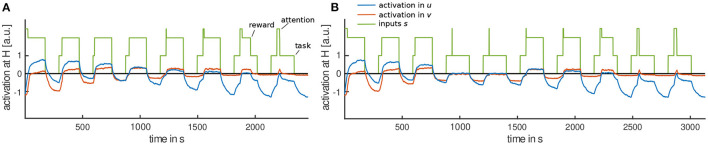
Exemplary time courses of activation until habituation criterion is met: **(A)** With reward input applied on all trials, the habituation criterion is met on the eighth trial. **(B)** When reward input is omitted in trials four to six, the habituation criterion is met on the tenth trial.

We expect that toddlers stop moving the lever when the rewarding outcome is suppressed, for example, by no longer playing the music. This was not tested in the experiment presented, but probed in the model by setting parameter values such that task input alone was not sufficient to cause a peak in the intention field. To test how the model behaves when the reward input is omitted in later trials, after stabilizing and destabilizing memory traces have already been built, we modified the simulation procedure. With all parameters of the model unchanged, the procedure was altered by switching off reward inputs in trials four and five. Figure 8B shows that movement time decreased on those trials. This is because lower levels of activation are more easily inhibited by the habituation field. Figure 9 compares the time courses of activation until the habituation criterion is met with reward input applied in all trials (a) and reward input omitted in trials four and five (b).

A more interesting question might be whether not receiving a rewarding outcome affects the process of habituation. We predict that trials without reward input do not contribute or contribute less to habituation than trials with a rewarding outcome. Habituation criterion would then be met at later trials. Model simulations support this idea: When reward input applied in all trials, the criterion is met after 7.9(±0.3) trials averaged over 50 simulations. When reward input is omitted on trials four and five, the criterion is met in the 10.0(±0.2) trial on average.

The model predicts that movement time is decreased in trials without a rewarding outcome. Due to less activation in the movement intention field those trials do not contribute or contribute less to habituation. Movement times are reinstated when the rewarding outcome is perceived again, which “resets” the process of habituation and, thus, the habituation criterion is met in later trials. Figure 9B shows that activation in the sixth trial is increased compared to activation when the reward input was applied in all trials (Figure 9A).

#### 3.3.4. Simulation of a Selection Task

In selection tasks, a transition from familiarity preference in early trials to a novelty preference in later trials is often observed. In the A-not-B paradigm, perseverative reaching could be viewed as a form of familiarity preference. The findings by Marcovitch et al. (2002) and Marcovitch and Zelazo (2006) show that with more experience of reaching to the A location, infants are less likely to perseverate. This could be viewed as a signature of habituation and a form of novelty preference.

Our experiment did not probe action selection. In the model, we may simulate action selection by simultaneously providing input at two field locations. This simulations can then be compared to the perseverative reaching paradigm. Task input is repeatedly applied to one field location, with a trial duration of 15s and an inter-trial period of 12s. In a second phase, an additional input over a second field location is added which competes with the continued input at the first location. This second phase occurs either early or late during habituation to the stimulus at the initial location.

Attention input is only applied in the first trial as it would bias the selection decision to one of the two movement directions. The familiar task input is applied at *x* = *H*, the additional input is applied at *x* = *V*. The amplitude of the novel task input, *s*_T_(V), is chosen such that it may induce a supra-threshold peak in the intention field. Therefore it is larger than the familiar task input *s*_T_(H). The parameter values of the model were left unchanged.

**Figure 10:**
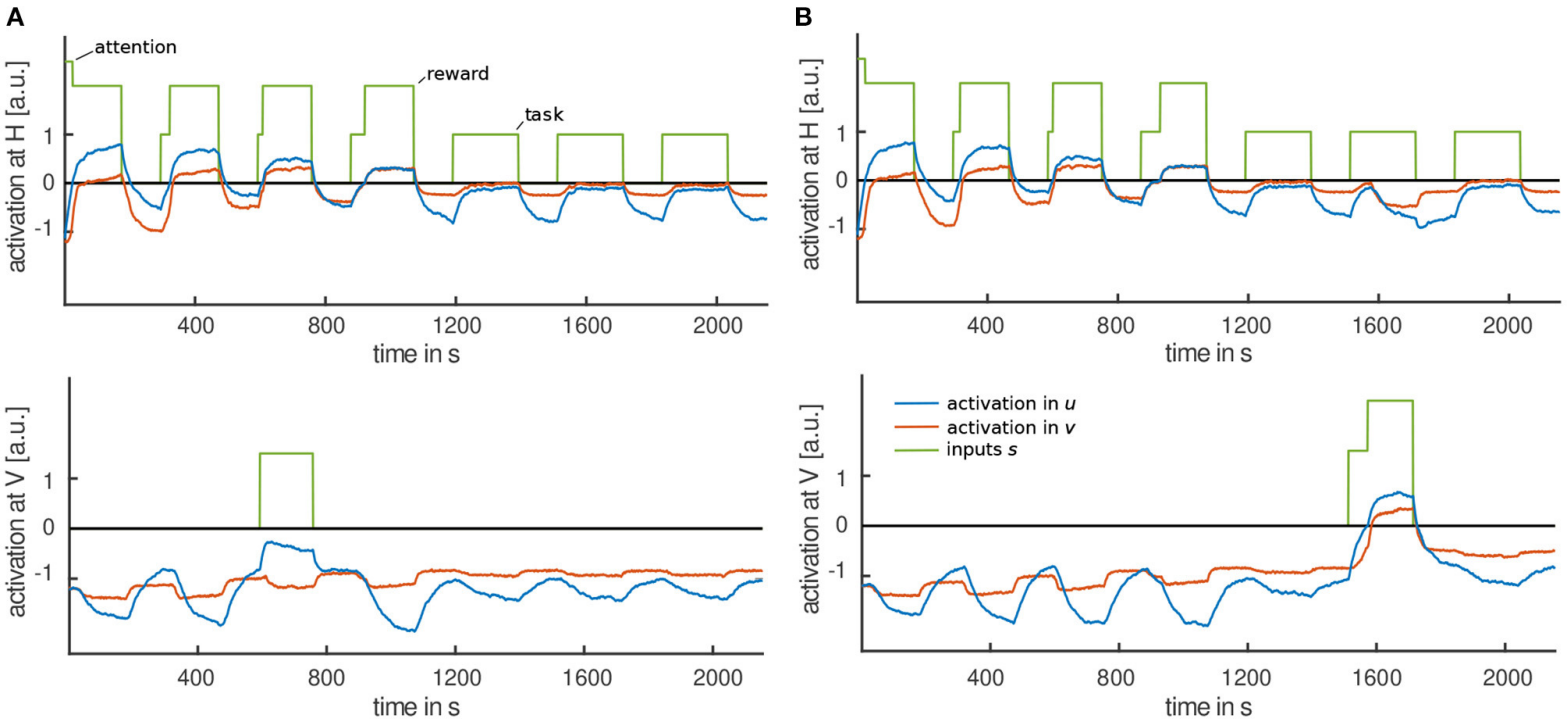
**(A)** Time courses of activation at the horizontal (top) and vertical (bottom) field locations with an additional stimulus applied at *x* = *V* on trial three. The stimulus at the familiar location (top) wins the selection decision. Activation at horizontal field location goes through the detection instability first and suppresses other field locations. **(B)** Same when the additional stimulus is applied on trial six. The stimulus at the novel location (bottom) wins the competition when activation becomes supra-threshold at vertical movement direction and other field locations are inhibited.

Figure 10 shows the resulting time courses of activation in the two fields. When the second stimulus input at *x* = *V* is applied on the third trial, activation at initial location, *x* = *H*, reaches positive values faster than at the novel location, despite the new input being larger than the familiar one. This is because peak formation at the familiar location is already facilitated by the stabilizing memory trace *u*_mem_ there (not shown). Because the intention field is selective, activation at *x* = *V* then remains sub-threshold (see Figure 10A). When the second stimulus input at *x* = *V* is applied on the tenth trial, activation at that new field location reaches the threshold faster than at the familiar location. This is because, at that familiar location, inhibition from the habituation field now predominates over the stabilizing memory trace (not shown). Once activation at *x* = *V* goes through the detection instability other field locations are inhibited and activation at *x* = *H* decreases (see Figure 10B).

In the A-not-B task, the selection decision to move to either the A or the B location is made on every trial. That selection is biased by the cue given to either location. Perseveration is measured as the preferred selection of the familiar movement even when the cue is given to the new movement. In the model, larger amplitude of the task input at *x* = *V* may be interpreted as the cue given to that movement direction. The simulation shows that the model produces the same pattern, an early preference of the familiar choice, a late preference of the novel choice. The model thus unifies an account for habituation and perseveration for movement tasks.

## 4. Discussion

We proposed a neural dynamic model that combines mechanisms previously postulated to explain perseverative motor behavior (Thelen et al., 2001; Dineva and Schöner, 2018) with mechanisms previously proposed to explain habituation to visual stimuli (Schöner and Thelen, 2006; Perone and Spencer, 2013b). This sets up an analogical mapping between the perceptual and motor domains. Perseveration in the motor domain corresponds to familiarity preference in the perceptual domain in that both are being caused by the build-up of activation in excitatory neural representations of movement parameters and of visual perceptions, respectively. The build-up of activation in an inhibitory layer of such a representation is the cause of habituation in the perceptual domain. Dishabituation and novelty preference result when a novel stimulus is presented after habituation has occurred to an earlier (familiar) stimulus. The analogical mapping predicts that similar effects of habituation and dishabituation should be observed in the motor domain. The mapping also predicts that novelty preference should be observed in the motor domain after habituation to a familiar movement.

We reported experimental evidence for the first part of this prediction. By applying the typical habituation paradigm to a motor task, we found a significant decrease of duration over which movements were performed and of the total movement path length during the habituation phase. When a new movement direction was enabled, we observed recovery of the movement time and path, an index of dishabituation. When the original movement direction was tested again, we observed signatures of Spencer-Thompson dishabituation. These results provide evidence for habituation in motor behavior that is specific to a particular movement, here probed by movement direction. We showed that the neural dynamic model accounts for all three signatures, habituation, dishabituation, and Spencer-Thompson dishabituation, through an approximate quantitative fit.

We provided theoretical evidence for the second prediction by simulating the model in a selection task. Activation was first induced for one value of the movement parameter by providing input at the corresponding location in the field. When this input was paired with an input at a competing location, the model selected the initial (familiar) location early during a sequence of habituation trials, but selected the second (novel) location late during the sequence of habituation trials. Mapped onto the A-not-B paradigm, the first pattern is consistent with perseveration after a small number of A trials (Wellman et al., 1986; Smith et al., 1999), the second pattern is consistent with reduced perseveration and enhanced switching to B after a larger number of A trials (Marcovitch et al., 2002; Marcovitch and Zelazo, 2006).

Together, the experimental and modeling results support a unified account in which motor behaviors and orientation responses are stabilized early during the experience of a motor behavior or a percept. With extended experience, the motor behavior or orientation response is destabilized, which promotes switching to alternate motor behaviors or re-orientation to alternate perceptual objects. This unified account is possible within the framework of Dynamic Field Theory because that framework postulates that all behaviorally significant neural states are attractors, whose stability prevents change. Transitions to new behavioral states are mediated by instabilities, the reduction of the attractors' stability. In DFT, enhanced stability comes from the accumulation of activation in excitatory populations that was modeled here by a memory trace, but that could also occur through the strengthening of synaptic connections from inputs to the excitatory populations. Conversely, reduced stability comes from the accumulation of activation in inhibitory population, likewise modeled by a memory trace here, but potentially taking the form of strengthening of synaptic connections from excitatory to inhibitory populations. The switch of activation state within the neural dynamic fields directly implements the decision to engage in a particular movement behavior or orientation response. Earlier work has established how such decisions can be directly coupled into a dynamics of fixation and gaze shift (Kopecz and Schöner, 1995; Perone and Spencer, 2013b) and into a dynamics of reaching movements (Schöner et al., 2019). In that respect, the account goes beyond earlier neural dynamic models that use overlapping ideas, in which levels of activation are mapped onto amounts of looking (Sirois and Mareschal, 2004) or probabilities of reaching to a location (Munakata, 1998).

The link between the build-up of excitatory/inhibitory activation and stability/instability offers a perspective on how processes of behavioral and perceptual exploration may be steered. This is a very broad topic that has been studied in many different settings. One notion that can be formalized mathematically (e.g., Kompella et al., 2017) is that “curiosity,” assigning high value to behaviors or state that create much variance, may structure the exploration of a state space. At a high level, this notion may appear compatible with a Sokolovian idea of investing into behaviors, while they are novel, and turning away from them, when they become known. In our much lower level account, such behavior is ultimately always directed at objects (Ruff, 1986), framed as perceptual outcomes or as the targets of movement behavior. By modeling the Sokolovian idea of “turning away from” as a destabilization of the ongoing behavior or orientation, the neural dynamic account suggests that exploration emerges as other objects or behaviors compete with a now destabilized earlier choice.

This raises the question, at which level this competition takes place. We looked only at a very low level of movement representations, the direction of a lever movement. Similarly, models of visual habituation have invoked very simple feature spaces, over which neural representations are built (Sirois and Mareschal, 2002; Schöner and Thelen, 2006). In reality, behavioral choices may be made at the levels of action goals (Raab and Hartley, 2018), potentially linked to the possible outcomes of such action (Herbort and Butz, 2012). Outcomes are perceptual events that occur once an action has been performed. Our account is far from reaching such a level, but it may be worthwhile to think through the implications for the concrete paradigms we modeled.

At what level may the movement decisions have been made in the experiment we reported? The effect of visual habituation was minimized, so we do not think that it is the visual appearance of the lever or the perception of hand's movement that matter. We also found that habituation did not depend on the hand used. So it is not likely, that the level of motor actions for particular effectors matters. The perceptual outcome of movement was the music that played in response to the toddler's movement. Unfortunately, the experiment did not probe the role of that outcome dimension. Informally, we observed that toddlers were not interested in moving the lever without perceiving the music. In the model, we tested how the omission of the reward input that models how the outcome affects the habituation process: Trials without reward input do not contribute or contribute less to habituation. The model predicts that the rewarding outcome of an action influences the intention to move and through that, the process of habituation. Analogously, the toy-less version of the A-not-B paradigm (Smith et al., 1999) shows that perseveration does not necessarily depend on knowledge about the hidden toy. Movements were motivated by attracting the infants attention to identical visible objects (lids) at the two locations. In this view, any outcome that is interesting enough to elicit a movement may impact on perseveration and habituation. A concrete task for future work would be to lift the ideas of stabilization and destabilization discussed in this article to the levels of goal and outcome representation, which would open goal selection and outcome prediction to neural dynamic accounts. Empirical support for such a generalization may come from the paradigm of voluntary task switching (Arrington and Logan, 2004).

## Data Availability Statement

The datasets presented in this article are not readily available because there is no agreement to share the data. Requests to access the datasets should be directed to gregor.schoener@rub.de.

## Ethics Statement

The studies involving human participants were reviewed and approved by Department of Psychology, Indiana University, Bloomington IN, USA. Written informed consent to participate in this study was provided by the participants' legal guardian/next of kin. Written informed consent was obtained from the participants' legal guardian/next for the publication of any potentially identifiable images or data included in this article.

## Author Contributions

JF and GS designed the experiment. JF performed the experiment and analyzed the data. SA and GS designed the model and wrote the manuscript. SA performed model simulations. All authors contributed to the article and approved the submitted version.

## Funding

This work was funded by the Deutsche Forschungsgemein-schaft (DFG, German Research Foundation)—402791869 (SCHO 336/12-1) within the SPP The Active Self (SPP 2134). We acknowledge support by the Open Access Publication Funds of the Ruhr-Universität Bochum.

## Conflict of Interest

The authors declare that the research was conducted in the absence of any commercial or financial relationships that could be construed as a potential conflict of interest.

## Publisher's Note

All claims expressed in this article are solely those of the authors and do not necessarily represent those of their affiliated organizations, or those of the publisher, the editors and the reviewers. Any product that may be evaluated in this article, or claim that may be made by its manufacturer, is not guaranteed or endorsed by the publisher.

## Footnotes

^1^How such intentional activation may actually drive movement generation down to activating muscles is not modeled here. See Schöner et al. (2019) for a sketch of such a more complete DFT model of the generation of reaching movements.

^2^see www.dynamicfieldtheory.org for access to the sources.
